# Park7 Expression Influences Myotube Size and Myosin Expression in Muscle

**DOI:** 10.1371/journal.pone.0092030

**Published:** 2014-03-17

**Authors:** Hui Yu, Jolena N. Waddell, Shihuan Kuang, Christopher A. Bidwell

**Affiliations:** 1 Department of Animal Sciences, Purdue University, West Lafayette, Indiana, United States of America; 2 Center for Cancer Research, Purdue University, West Lafayette, Indiana, United States of America; Tohoku University, Japan

## Abstract

Callipyge sheep exhibit postnatal muscle hypertrophy due to the up-regulation of *DLK1* and/or *RTL1*. The up-regulation of *PARK7* was identified in hypertrophied muscles by microarray analysis and further validated by quantitative PCR. The expression of PARK7 in hypertrophied muscle of callipyge lambs was confirmed to be up-regulated at the protein level. PARK7 was previously identified to positively regulate PI3K/AKT pathway by suppressing the phosphatase activity of PTEN in mouse fibroblasts. The purpose of this study was to investigate the effects of PARK7 in muscle growth and protein accretion in response to IGF1. Primary myoblasts isolated from *Park7* (+/+) and *Park7* (−/−) mice were used to examine the effect of differential expression of *Park7*. The *Park7* (+/+) myotubes had significantly larger diameters and more total sarcomeric myosin expression than *Park7* (−/−) myotubes. IGF1 treatment increased the mRNA abundance of *Myh4, Myh7 and Myh8* between 20-40% in *Park7* (+/+) myotubes relative to *Park7* (−*/*−). The level of AKT phosphorylation was increased in *Park7* (+/+) myotubes at all levels of IGF1 supplementation. After removal of IGF1, the *Park7* (+/+) myotubes maintained higher AKT phosphorylation through 3 hours. PARK7 positively regulates the PI3K/AKT pathway by inhibition of PTEN phosphatase activity in skeletal muscle. The increased PARK7 expression can increase protein synthesis and result in myotube hypertrophy. These results support the hypothesis that elevated expression of *PARK7* in callipyge muscle would increase levels of AKT activity to cause hypertrophy in response to the normal IGF1 signaling in rapidly growing lambs. Increasing expression of PARK7 could be a novel mechanism to increase protein accretion and muscle growth in livestock or help improve muscle mass with disease or aging.

## Introduction

Callipyge sheep exhibit postnatal muscle hypertrophy, with higher rates of protein accretion and lower rates of fat deposition compared to normal sheep [1], [2]. The muscle hypertrophy phenotype is most prominent in the loin and hind-quarters at 4–6 weeks of age due to increased muscle fiber diameter and percentage of fast-twitch, glycolytic muscle fibers, [3]–[6]. The callipyge mutation is a single nucleotide polymorphism in the *DLK1-DIO3* imprinted gene cluster [7], [8] that causes up-regulation of *Delta-like 1 (DLK1)* and *Retrotransposon-like 1 (RTL1)* in hypertrophied muscles [9]–[13]. Transgenic mice over-expressing *Dlk1* exhibited increased muscle mass and myofiber diameter [14]. Muscle-specific gene ablation of *Dlk1* in the mouse resulted in reduced body weight and skeletal muscle mass due to reductions in myofiber numbers [15]. Conversely, over-expression of *Dlk1* in culture was shown to inhibit myoblast proliferation and enhance myotube differentiation [15].

Microarray analysis of gene expression identified 199 genes that were differentially expressed in *semimembranosus* muscle of callipyge and normal lambs [16]. *Parkinson Protein 7 (PARK7;* also known as *DJ-1)* expression was up-regulated in hypertrophied muscles. *PARK7* encodes a ubiquitously expressed, highly conserved protein that has been shown to be involved in diverse biological processes including oxidative stress response, transcriptional regulation and cell survival modulation. A mutation causing a loss of function of *PARK7* was found to be responsible for a recessive early-onset form of Parkinson's disease [17]. PARK7 protects neurons and somatic cells from oxidative stress by oxidizing itself to a more acidic form [18]. PARK7 enhances the NF-κB pathway by binding to Cezanne [19], restores androgen receptor transcription activity by binding to PIAS1 (protein inhibitor of activated STAT,1) [20], and up-regulates human tyrosine hydroxylase gene expression by interaction and inhibition of PSF (Polypyrimidine tract-binding protein-associated splicing factor) [21]. *Park7* was originally identified as an oncogene that transforms NIH3T3 cells in cooperation with the activated *Ras* gene [22]. Later, several studies have shown that PARK7 is involved in the progression of many cancers [23]–[28]. The mechanisms involve PARK7 binding to p53BP3, p53 [29], [30], DAXX (death domain-associated protein), ASK1 (Apoptosis signal-regulating kinase 1) [31], [32] and PTEN (Phosphatase with tensin homology) [33] to regulate cell cycle progression. PARK7 was shown to suppress the phosphatase activity of PTEN which is a negative regulator of the phosphatidylinositol 3′ kinase (PI3K)/AKT pathway [33]–[35]. The phosphorylation of AKT activates several pathways to regulate cell proliferation [36], cell survival [37] and protein synthesis [38].

The PI3K/AKT pathway is known to positively regulate muscle growth [39], [40]. The binding of insulin-like growth factor 1 (IGF1) to its receptor initiates this pathway and activates AKT. Addition of IGF1 into culture medium induced hypertrophy in C2C12 myotubes through enhanced activation of AKT [40]. Muscle-specific over-expression of *Igf1* caused muscle hypertrophy in mice [41] and conversely muscle-specific inactivation of the *Igf1* receptor impaired muscle growth due to reduced muscle fiber number and size [42]. It also had been well demonstrated that the activation of AKT is sufficient to induce hypertrophy. Over-expression of activated *Akt* in muscle fibers results in significantly larger fiber size [39], [43]. Transgenic mice expressing a constitutively active form of *Akt* in muscle exhibit rapid skeletal muscle hypertrophy [44]. Conversely, genetic depletion of *Akt* in mice leads to a smaller body size and shorter life span [45].

Though extensively studied, a role for PARK7 in muscle growth had not been reported until recently. In addition to callipyge lambs [16], transcriptomic and proteomic analyses in quadriceps muscles in myostatin-null mice, which exhibit a muscle hyperplasia phenotype, found elevated levels of PARK7 and phosphorylation of AKT [46]. Further work by the same group in double-muscled cattle also showed higher PARK7 expression in double-muscled fetuses compared to normal controls [47]. In food animal agriculture, there is a need to identify genes that can increase muscle growth and protein accretion. The identification of elevated expression of *PARK7* in hyper-muscular animals suggests a hypothesis that increasing *PARK7* gene expression can increase muscle growth and protein accretion. Differential expression of PARK7 was examined in this study using *Park7* (+/+) and *Park7* (−/−) mouse models to investigate the effects of *Park7* gene expression in muscle growth and protein accretion in response to IGF1.

## Results

### PARK7 is up-regulated in hypertrophied muscle of callipyge lambs

**Figure 1:**
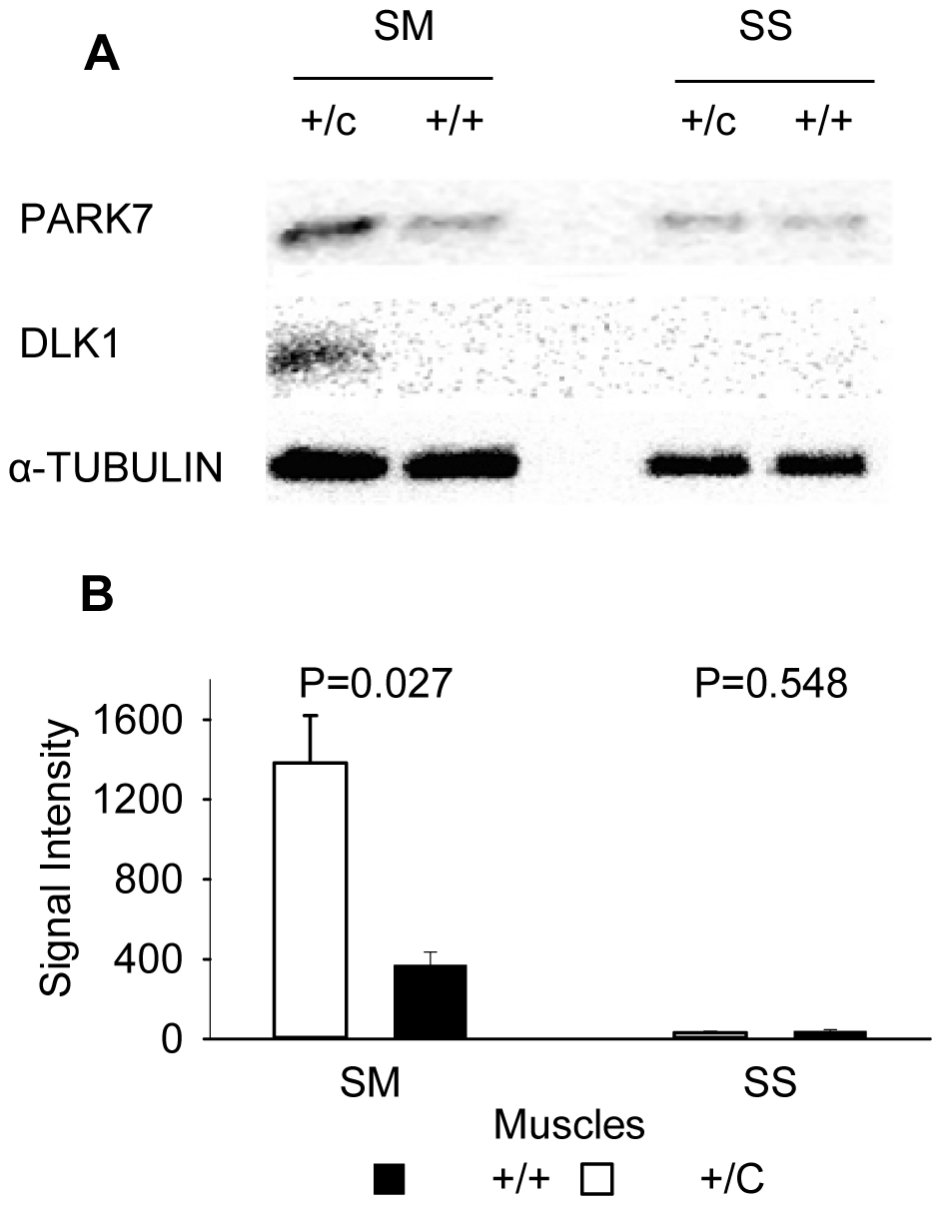
PARK7 protein expression level in sheep skeletal muscles. A: There were higher levels of PARK7 and DLK1 proteins in the semimembranosus (SM) of callipyge lambs (+/*C*) relative to normal lambs (+/+), but no difference in PARK7 or DLK1 protein levels in the supraspinatus (SS). B: Quantitative analysis of PARK7 protein expression was significantly higher in the SM of callipyge lambs. α-Tubulin was the control for protein loading. Data shown are mean signal intensity ± SE for the PARK7 without normalization using three animals per genotype. The p-values for comparisons between genotypes for each muscle are shown.

*Semimembranosus* and *supraspinatus* muscle samples were collected from 3 callipyge and 3 normal lambs at 30–35 days of age when muscle hypertrophy is detectable by muscle mass. The *semimembranosus* from the pelvic limb undergoes the largest magnitude of hypertrophy and *supraspinatus* from the thoracic limb does not become hypertrophied [1], [48]. Western blots (Figure 1A) showed that PARK7 protein expression was elevated about 3-fold (P = 0.027; Figure 1B) in the *semimembranosus* of callipyge (+/C) relative to normal (+/+) lambs. There were no differences between genotypes in the *supraspinatus*. Elevated levels of DLK1 protein were also confirmed in the callipyge *semimembranosus*. No detectable DLK1 expression was found in the muscles of normal animals and non-hypertrophied muscles, along with a reduced PARK7 expression in these same animals and tissues.

### Effects of *Park7* genotype on myosin heavy chain gene expression

The initial *Park7* (−/−) mouse was obtained from Jackson Laboratory and back crossed with C57BL6 mice to generate *Park7* (+/+), (+/−) and (−/−) littermate mice. Live animal body weights were monitored and collected from 3 – 6 weeks of age. Mice were euthanized at 6 weeks and carcass (muscle and skeleton) weight and internal organs weights were collected. The phenotypic data from male and female mice was analyzed by linear regression separately to account for gender differences. No significant differences were found between the three genotypes for analysis of live weight by age, carcass weight by live weight, or organ weight by live weight analysis in males or females (Figure S1 and Table S1).

**Figure 2:**
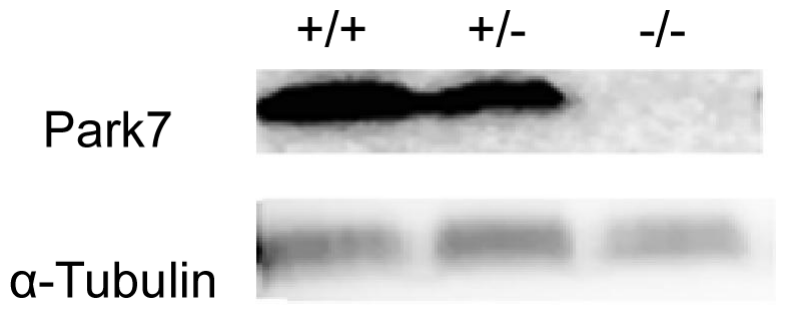
PARK7 protein expression in *vastus lateralis*. PARK7 protein expression was not detectable in *vastus lateralis* in *Park7* (−/−) mice in two replicated experiments of two animals per genotype. α-Tubulin was used as a control to show equal protein loading.

The expression of PARK7 protein in the *vastus lateralis* muscle was examined to verify the loss of expression in *Park7* (−/−) mice (Figure 2). The expression of PARK7 was easily detectable in the muscles in *Park7* (+/+) and *Park7* (+/−) animals, but no PARK7 protein was detectable in *Park7* (−/−) muscles.

**Figure 3:**
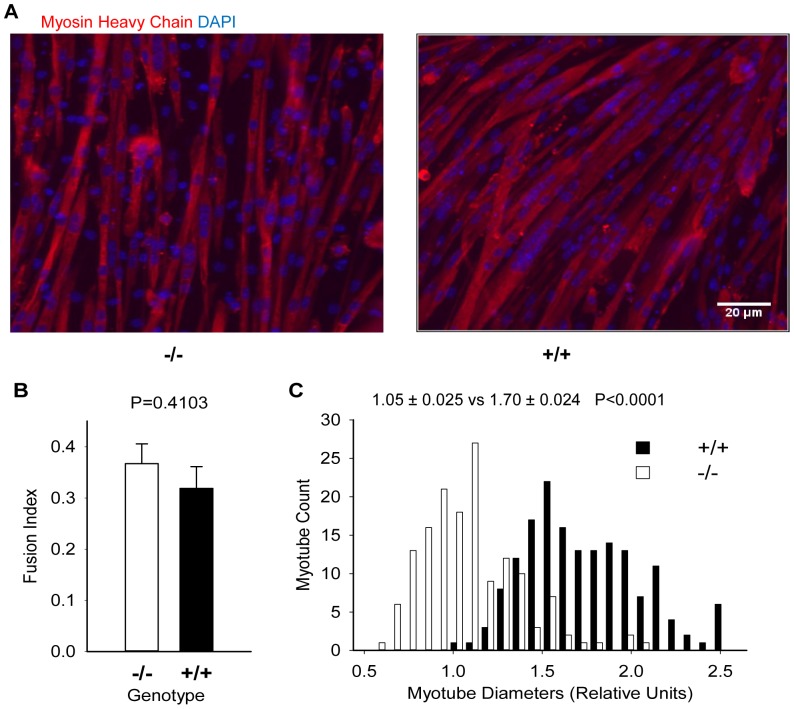
Comparison of myotube size between *Park7* (−/−) and *Park7* (+/+) genotypes. A: Primary myoblasts were induced to differentiate for 72 hours and stained with an antibody for total myosin heavy chain (red: MF20) and DNA (blue: DAPI). B: No significant differences were detected between the two genotypes for fusion index. C: The distributions of myotube diameters for the two genotypes are shown. The mean diameter for *Park7* (+/+; 1.70±0.24) myotubes were significantly larger (P<.0001) than *Park7* (−/−; 1.05±0.025) myotubes.

**Figure 4:**
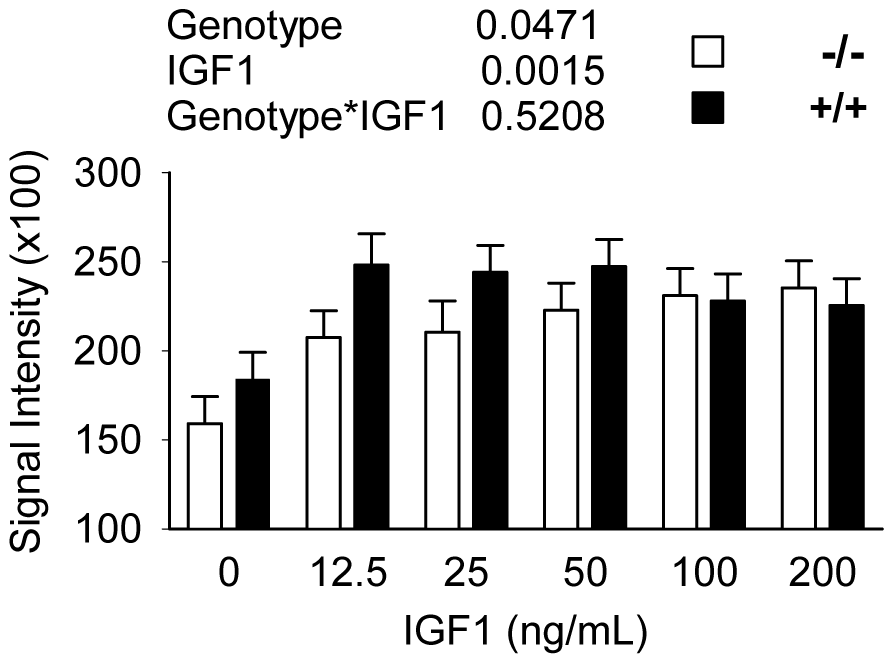
Detection of total sarcomere myosin expression in myotubes by ELISA. There were significant effects for genotype and IGF1 treatment on total myosin, but there was no significant interaction. The *Park7* (+/+) myotubes had significantly more total sarcomere myosin expression than *Park7* (−/−) myotubes and addition of IGF1 at all concentrations induced significant higher sarcomere myosin compared to no added IGF1.

Primary myoblasts were isolated from *Park7* (+/+) and *Park7* (−/−) animals and fused into myotubes in order to model the effect of *Park7* levels on growth and myosin expression. The relative sizes of myotubes were determined after 72 hours of differentiation in fusion media (Figure 3A). The fusion index was calculated to determine the proportion of myoblasts that fused into myotubes, and myotube diameters were measured to estimate relative myotube size. There were no differences (p = 0.4103) in the fusion index between the *Park7* (−/−) and (+/+) cultures (Figure 3B). *Park7* (+/+) myotubes were significantly larger (1.6-fold; p<0.0001) than *Park7* (−*/*−) myotubes (Figure 3C). An *in vitro* ELISA assay was performed to detect total sarcomeric myosin protein accretion to test the hypertrophy mechanism. IGF1 treatments were included due to its well-established role in regulating muscle hypertrophy [39], [40]. Two-way analysis of variance indicated the overall effect of genotype was significant (p = 0.0471) with the *Park7* (+/+) myotubes having about 10% higher myosin expression than *Park7* (−/−) myotubes, regardless of IGF1 treatment (Figure 4). All IGF1 treatments had a significant effect compared to no added IGF1 (p = 0.0015), with 34% higher myosin expression in IGF1 treated myotubes. The genotype by IGF1 interaction effect was not significant (p = 0.5208) but the effect IGF1 treatment appeared to reach saturation at lower concentrations for *Park7* (+/+) myotubes (25 ng/mL) relative to *Park7* (−/−) myotubes (100 ng/mL).

**Figure 5:**
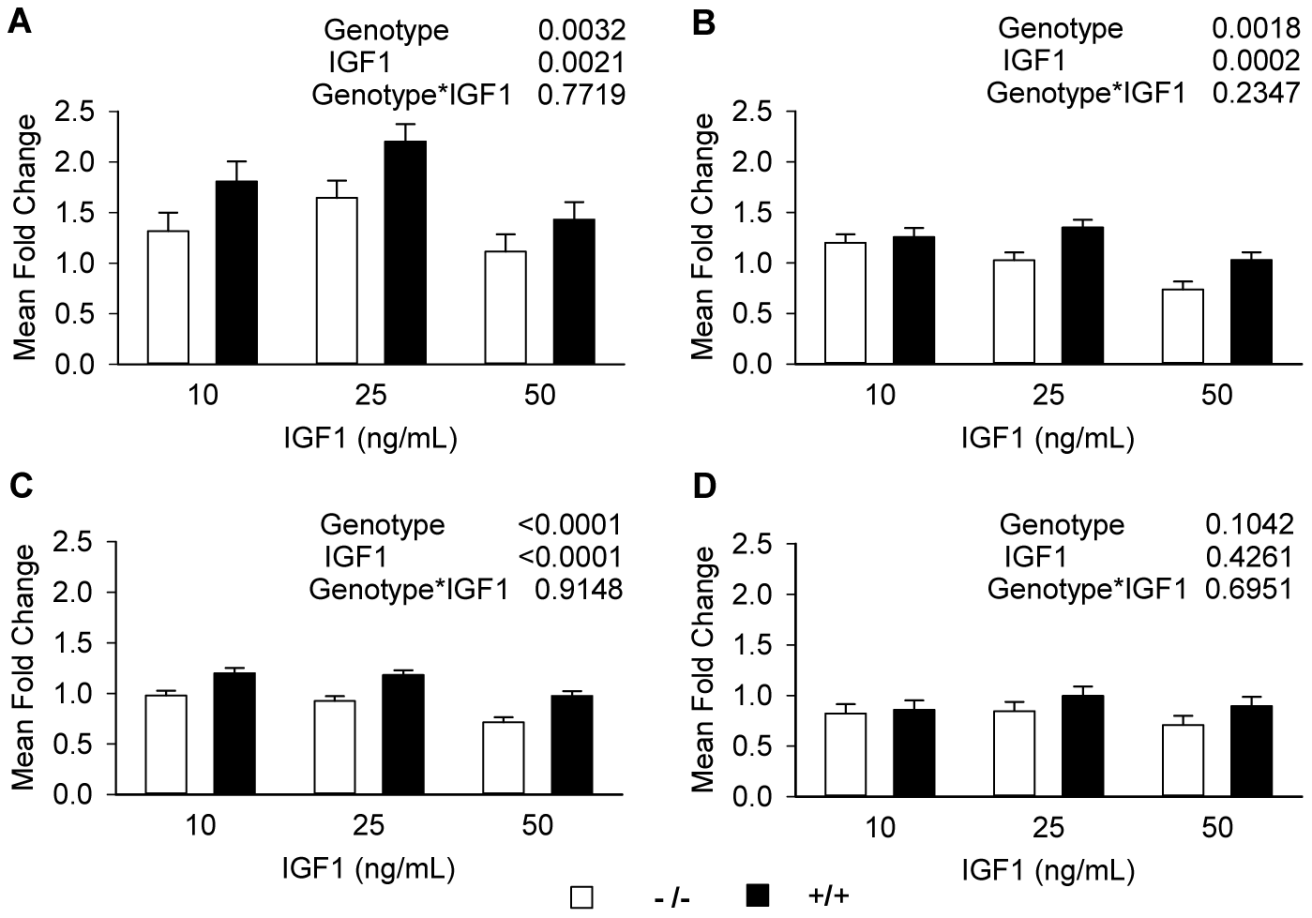
Detection of myosin heavy chain isoform mRNA levels in myotubes by quantitative PCR. Primary myoblasts were isolated from 2 animals for each genotype and two dishes of cells from each animal (4 replicates) were used for each genotype by IGF1 treatment combination. IGF1 was added to the culture 24 hours after differentiation at the indicated concentration. A: *Myh4 (Myosin Heavy Chain, Type IIB)* expression; B: *Myh7 (Myosin Heavy Chain, Type I)* expression; C: *Myh8 (Myosin Heavy Chain, perinatal)* expression; D: *Myh3* expression *(Myosin Heavy Chain, embryonic)*. *Myh4*, *Myh7* and *Myh8* had significantly higher expression in *Park7* (+/+) myotubes, however, no significant differences were detected for *Myh3*. The mean fold change value was calculated using the 2 ^–ΔΔCt^ method with *Rplp38* as the control gene and IGF1 concentration of 0 ng/mL as the control treatment.

Quantitative PCR was used to detect the effect of *Park7* genotype on *Myh4*, *Myh7*, *Myh8* and *Myh3* expression in IGF1 treated mature myotubes (Figure 5). The mRNA abundance of *Myh4* was 40% greater (p = 0.0032) in *Park7* (+/+) myotubes, relative to *Park7* (−/−) myotubes (Figure 5A). The addition of IGF1 in culture induced significantly higher *Myh4* expression with 1.6-fold increase at 10 ng/mL, 1.9-fold increase at 25 ng/mL and 1.3-fold increase at 50 ng/mL of IGF1 respectively. The *Myh7* and *Myh8* genes (Figure 5 B and C respectively) had similar expression patterns as *Myh4* but with smaller changes in gene expression. The mRNA abundance of *Myh7* and *Myh8* were increased 20% (p = 0.0018) and 30% (p = 0.0001) respectively in *Park7 (+/+)* myotubes. IGF1 also stimulated *Myh7* and *Myh8* mRNA expression, but only at the lower concentration (10 ng/mL) with 23% increase in *Myh7* and 10% increase in *Myh8* respectively, relative to the treatment without IGF1. At the high concentration of IGF1 (50 ng/mL), *Myh7* expression dropped 12% and *Myh8* expression dropped 16% compared to the treatment without IGF1. *Myh3* was the only isoform measured that showed no genotype effect or IGF1 effect. There was no significant genotype by IGF1 interactions for the four myosin isoforms.

### Effect of *Park7* genotype on phosphorylation level of AKT (S473)

**Figure 6:**
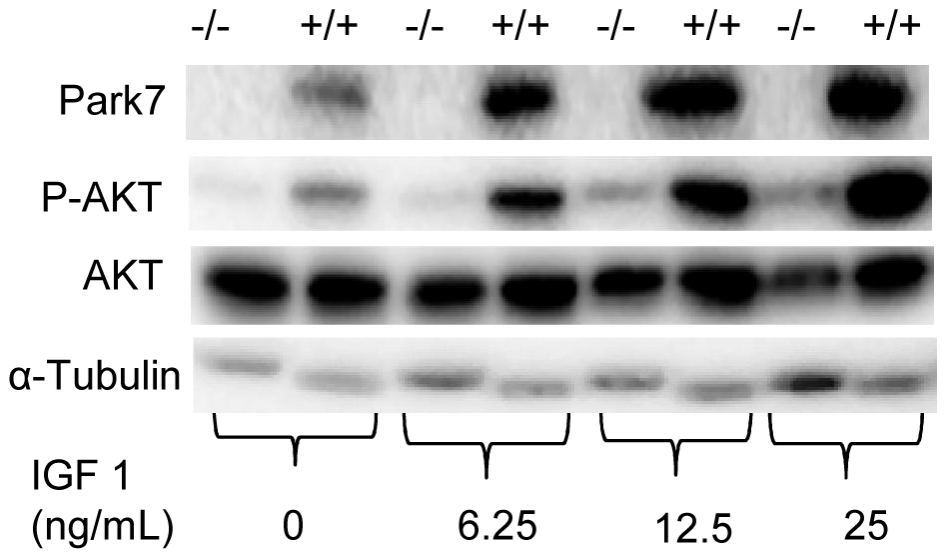
Phosphorylation of AKT (S473) protein in *Park7* (+/+) and *Park7* (−/−) myotubes. Myoblasts were induced to differentiation for 72 hours and treated with IGF1 at the indicated concentrations for 15 minutes. There was increased phosphorylation of AKT (P-AKT) in *Park7* (+/+) myotubes relative to total AKT (AKT) in myotubes in fusion medium (5% horse serum). There was a substantial increase in AKT phosphorylation in *Park7* (+/+) myotubes relative to *Park7* (−/−) myotubes at all IGF1 concentrations. α-Tubulin was the control for protein loading.

**Figure 7:**
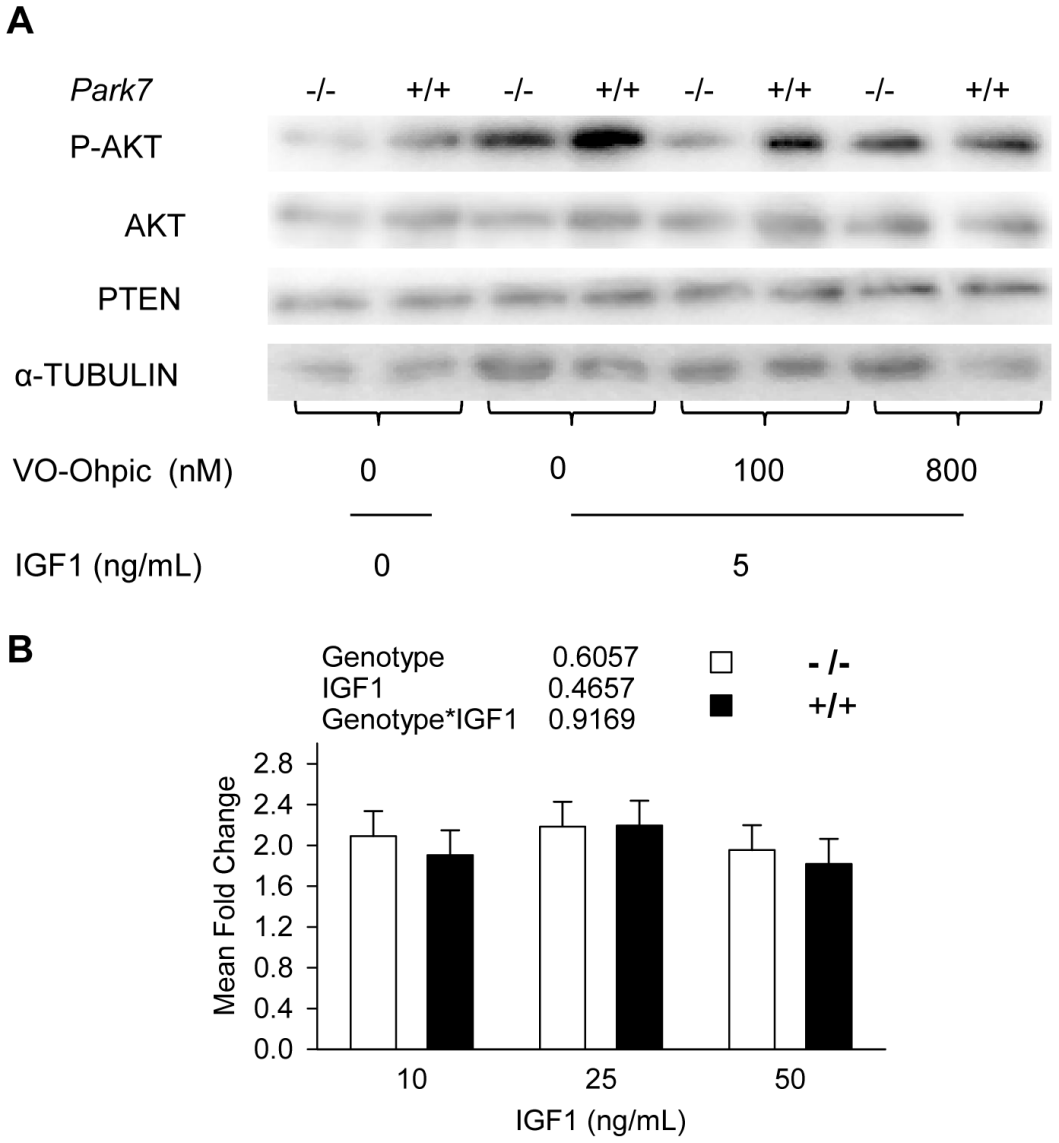
PARK7 Inhibits PTEN function, but not PTEN expression. A: Western blot analysis of phosphorylated AKT (S473) in *Park7* (+/+) and *Park7* (−/−) myotubes in the presence of different concentrations of PTEN inhibitor (VO-OHpic) after IGF1 stimulation. Differential phosphorylation of AKT due to *Park7* genotype was eliminated by the PTEN inhibitor VO-Ohpic at 800 nM. No differences were detected in PTEN expression. α-Tubulin was the control for protein loading B: Detection of *Pten* mRNA level in myotubes by quantitative PCR. No significant differences were detected between the two genotypes or IGF1 treatment. The mean fold change was calculated using the 2 ^–ΔΔCt^ method with *Rplp38* as the housekeeping gene and no added IGF1 as the control treatment.

In order to explore the mechanism of *Park7* regulation on myotube size and myosin gene expression, a series of experiments were conducted to assess the activation of AKT by phosphorylation at S473 in presence or absence of *Park7*. It has been demonstrated that PARK7 can suppress PTEN phosphatase activity, which would have the effect of increasing receptor tyrosine kinase signaling through AKT [33]. IGF1 was used to test the effect of *Park7* genotype on the phosphorylation of AKT. The level of phosphorylation of AKT was elevated in *Park7* (+/+) myotubes relative to *Park7* (−/−) myotubes in 5% horse serum differentiation media without added IGF1 (Figure 6). The phosphorylation of AKT was substantially elevated in *Park7* (+/+) myotubes at all levels of added IGF1. To investigate if the activity of PARK7 could be mimicked by inhibition of PTEN function, VO-OHpic trihydrate, a known effective PTEN inhibitor [49] was introduced in this study. Myotubes were first treated with IGF1 (5 ng/mL) for 15 minutes to induce increased phosphorylation of AKT. Different concentrations of VO-OHpic trihydrate were then added for an additional 15 minutes after removing IGF1. The addition of VO-OHpic trihydrate at 800 nM increased the level of AKT phosphorylation in *Park7* (−/−) myotubes and eliminated the differences between the two *Park7* genotypes (Figure 7A). There were no discernable differences in PTEN protein levels (Figure 7A) or RNA expression (Figure 7B, p =  0.6057) between *Park7* (+/+) and *Park7* (−/−) myotubes.

**Figure 8:**
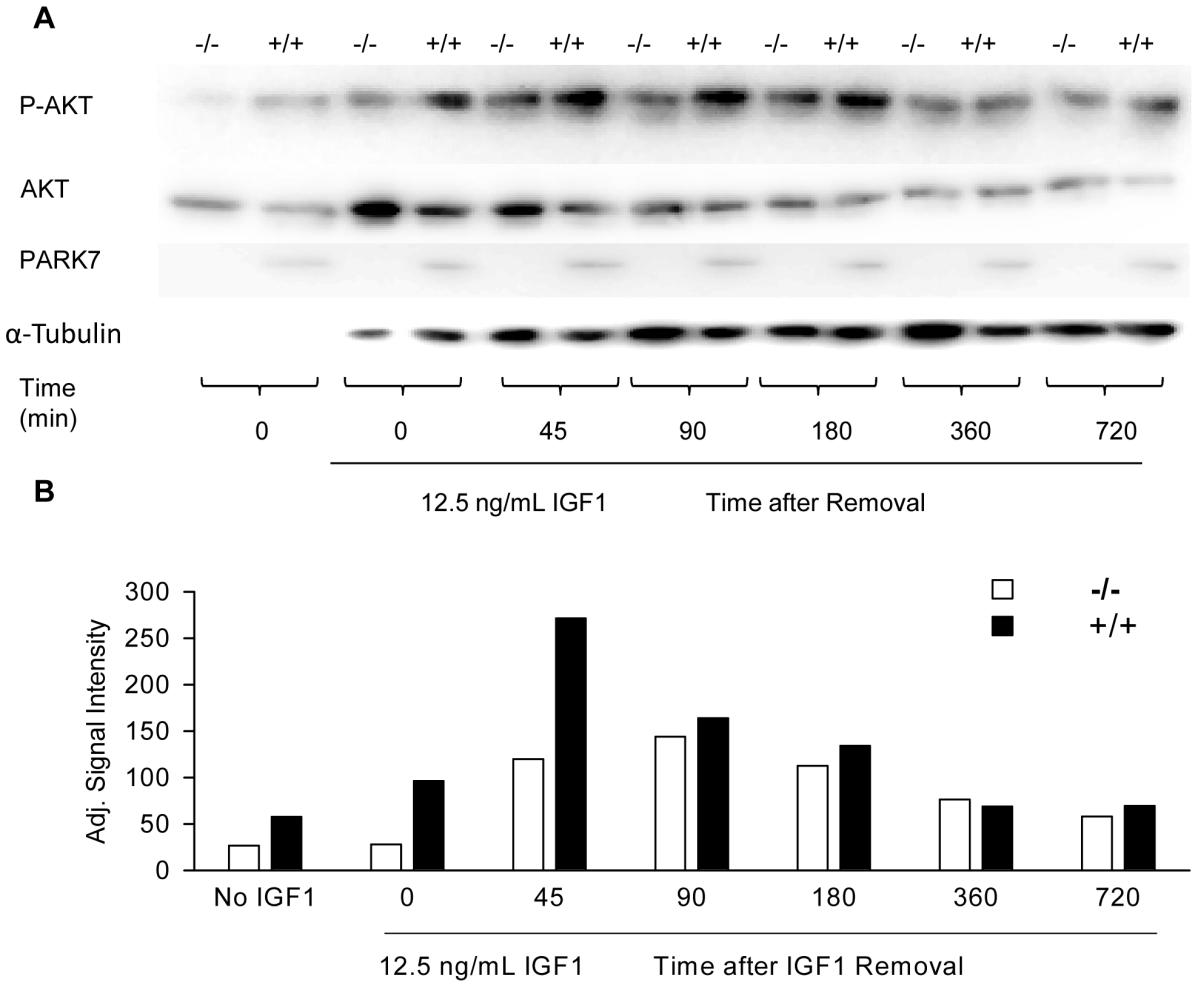
Sustained activity of AKT phosphorylation (S473) in *Park7* (+/+) and *Park7* (−/−) myotubes after IGF1 stimulation. Mature myotubes were stimulated with IGF1 (12.5 ng/mL) for 15 minutes. Total protein was extracted at the indicated time after removal of IGF1. A: Western blot analysis showed a higher level of AKT phosphorylation in *Park7* (+/+) relative to *Park7* (−/−) myotubes. PARK7 blotting confirmed the cell genotypes and α-tubulin blotting was used as a loading control. B: Analysis of the signal intensity for P-AKT/total AKT ratio (adj. signal intensity) showed the *Park7* (+/+) myotubes had higher AKT phosphorylation without added IGF1 and had a 3 fold higher level of P-AKT/total AKT than *Park7* (−/−) myotubes at 45 minutes after IGF1 treatment. The decline in AKT phosphorylation was similar between the genotypes after 90 minutes with *Park7* (+/+) myotubes generally maintaining approximately 20% higher signal intensity. This experimental design was replicated twice on individual blots with similar results. The signal intensity data was derived from the blot shown in A.

Since increased phosphorylation of AKT was found in *Park7* (+/+) myotubes, the timing and duration of the elevated phospho-AKT was tested. Myotubes were treated with IGF1 for 15 minutes and protein was collected at several time points up to 12 hours. Western blots showed increased phosphorylation of AKT in *Park7* (+/+) myotubes (Figure 8A). Quantitative analysis of relative signal intensity based on the ratio of phospho-AKT to total AKT indicated a 2-fold higher level of P-AKT in *Park7* (+/+) myotubes relative to *Park7* (−/−) myotubes with no added IGF1 (Figure 8B). The level of AKT phosphorylation in *Park7* (+/+) myotubes reached its maximum at 45 minutes at 3-fold higher signal intensity than *Park7* (−/−) myotubes after removal of IGF1. *Park7* (−/−) myotubes had maximum phosphorylation at 90 minutes and the decreases in phosphorylation over time were similar with *Park7* (+/+) myotubes, generally maintaining approximately 20% higher signal intensity through 12 hours.

## Discussion

Overall, the present results indicate that differential expression of the PARK7 protein can result in increased myosin gene expression, protein accretion and myotube growth through an altered response to IGF1/AKT signaling. This study focused on Park7′s effect on AKT phosphorylation due to its prominent role in regulating muscle growth, other reported biological activities in oxidative stress, anti-apoptosis and several regulatory pathways [17]–[21], [29]–[32] may also contribute to improved muscle growth

The *Park7* (−/−) mice are viable and lack obvious developmental abnormalities. No differences were found between *Park7* (+/+) and (−/−) animals *in vivo* when comparing body weight, carcass weight and internal organ weight which indicate that Park7 is not a dominant regulator of live animal growth. These results were consistent with other findings, which indicated no significant differences in body weights between the two genotypes from birth to 12 months [50]. Skeletal muscle growth in live animals is stimulated by several hormones such as IGF1, androgens and β-adrenergic agonists to initiate multiple signaling pathways to regulate growth.

A previous study showed elevated *PARK7* expression in callipyge sheep at the transcriptional level [16] and the present results confirmed the increased expression of PARK7 in hypertrophied muscles at the protein level. An *in vitro* cell culture model was used in this study to investigate the effects of differential expression of *Park7* on myogenesis. The *in vitro* comparison of myotube size showed *Park7* (+/+) myotubes had a bigger size and higher myosin expression than *Park7* (−/−) myotubes, but no significant change in the fusion index, which indicates *Park7* expression affects myotube hypertrophy (increased myofiber diameter), not hyperplasia (increased myofiber number). These results support the proposed models that elevated PARK7 expression is part of the physiological mechanism for increased muscle growth in callipyge lambs and myostatin null mice and cattle [16], [46], [47].

The PI3K/AKT pathway is one of the primary targets of IGF1 signaling and has been well characterized for its positive regulation on muscle growth [39], [40]. The postnatal muscle mass and myofiber size generally reflects protein accretion. AKT stimulates protein synthesis by activating mammalian target of rapamycin (mTOR) and its downstream effectors. This study showed that the *Park7* (+/+) myotubes had increased phosphorylation of AKT compared to *Park7* (−/−) after IGF1 stimulation, which induced significantly more total sarcomeric protein accretion. Notably, this increase of protein accretion reached saturation at lower IGF1 concentrations in *Park7* (+/+) relative to *Park7* (−/−) myotubes. The expression of *Myh4* showed the largest increases although *Myh7* and *Myh8* mRNA expression were also elevated in *Park7* (+/+) myotubes indicating that both myosin transcription and translation were affected by *Park7* expression. Muscle contractile characteristics partially depend on the expression of myosin heavy chain isoforms. The hypertrophied muscles in callipyge sheep have an increased size of fast twitch glycolytic myofibers along with increased *MYH4* expression and a decreased frequency of oxidative fibers [4], [6]. Over-expression of constitutively active *Akt* had been reported to promote myofiber growth with no effect on fiber specification in regenerating skeletal muscle [43]. The use of a doxycycline-inducible *Akt* transgene in mice resulted in the hypertrophy of glycolytic myofibers, but not oxidative myofibers [51]. A recent study supported this result by identifying a regulatory cascade of AKT activation to drive the metabolic and contractile specification of fast-twitch muscle fibers [52]. Similarly, the results presented here are congruent with the up-regulation of *Myh4* and the enhanced activation of AKT in the presence of *Park7*. Interestingly, double-muscled cattle, in which the expression of *Park7* gene is also elevated, have an increased proportion of fast-twitch glycolytic fibers as well [53].

The differences in phosphorylation of AKT in *Park7* (+/+) and *Park7* (−/−) myotubes was eliminated when treated with higher concentrations of PTEN inhibitor, but the total amount of PTEN had not been changed, suggesting PARK7 acts through inhibition of PTEN phosphatase activity, not total PTEN expression. This result was consistent with the findings of Kim *et al*. [33], [54]. In their studies, they also examined the phosphorylation level of PTEN at S380, T382, T383 and S385 in *Park7* transfected NIH3T3 cells, but no change was detected; indicating the inhibition of PTEN phosphatase activity by PARK7 is not affected by phosphorylation of PTEN. The mechanism by which PARK7 inhibits PTEN phosphatase activity remains unclear. Some studies have proposed that PARK7 can directly bind to PTEN to confer an inactive conformation by forming a disulfide bond between C71and C124 of PTEN upon oxidative stress [54]–[56]. It has also been speculated that the interaction of PARK7 with PTEN may accelerate the oxidation of PTEN, thus inactivating the PTEN function [54]. Further analysis will be necessary to determine how PARK7 inhibits PTEN function in muscle.

## Conclusions

The present study provides a novel insight into a role for *Park7* in the regulation of skeletal muscle growth. *Park7* expression can modulate IGF1/AKT signaling, increase myosin gene expression and protein synthesis and increase myotube size. These results support the hypothesis that elevated expression of *PARK7* is part of the physiological mechanism for muscle hypertrophy in callipyge lambs and increased muscle mass in double muscled cattle. Elevated expression of PARK7 in the muscles of callipyge lambs could lead to increased muscle growth in response to the normal IGF1 signaling present in young growing lambs. The prominent increase in *Myh4* gene expression in myotubes was also consistent with changes in myosin fiber types in callipyge muscle. *DLK1* is one of the causative genes for the callipyge phenotype due to the inheritance of the mutation [9], [12]; therefore elevated *PARK7* expression could be considered a downstream response to *DLK1* signaling. Identifying how *PARK7* gene expression is regulated in callipyge induced muscle hypertrophy could provide new mechanisms to increase livestock muscle growth and production efficiency or prevent the loss of muscle mass due to disease or aging.

## Materials and Methods

### Ethics Statement

Sheep were maintained at the Purdue Animal Sciences Research and Education Center under standard agricultural housing and management. Lambs were euthanized for tissue sampling using three lethal procedures of captive bolt stunning, exsanguination and pneumothorax. This procedure was approved by Purdue Animal Care and Use Committee (protocol #1112000493). All mice were fed with standard chow food and water, and housed at 22–23°C with nesting material with 12 hour light/dark cycle. Cage bedding was changed weekly by professional personnel. Mice were euthanized by cervical dislocation and pneumothorax for dissection of muscle tissue for the growth trial and a source of primary satellite cells. Mice housing and euthanasia was performed according to protocols approved by the Purdue Animal Care and Use Committee (protocol #1112000440).

### Mouse Growth Trial

Mice with the *Park7* locus inactivated by gene targeting [*Park (−/−)*] are available from JAX mice® (Strain 006577, Jackson Laboratories, Bar Harbor, ME). The initial C57BL6 *Park7* (*−*/*−*) mouse was crossed with C57BL6 *Park7* wild type (+/+) to produce heterozygous (+/*−*) *Park7* mice for breeders. Matings of heterozygous *Park7* mice were conducted to generate litters of mice with all three possible Park7 genotypes (+/+) (+/*−*) and (*−*/*−*). Twenty females with 4 *Park7* (+/+), 12 (+/*−*) and 4 (*−*/*−*) animals and twenty-five males with 4 *Park7* (+/+), 11 (+/*−*) and 10 (*−*/*−*) from seven litters were used to analyze live animal growth. Live animal weights were measured at 6 weeks of age to the nearest 0.01 g. The carcass and internal organ weights were measured on a total of 18 males (4 *Park7* (+/+), 6 (+/*−*) and 8 (*−*/*−*) animals) and 16 females (4 *Park7* (+/+), 8 (+/*−*) and 4 (*−*/*−*) animals) to the nearest 0.0001 g. Carcass weights were measured by removal of head, tail, skin and fur, internal organs and visceral fat of the mouse. The general linear model and genotype specific models for regression analysis are given in Supplementary Table S1.

### RNA Isolation and Quantitative PCR Analysis

RNA from cultured cells was extracted and purified using Nucleo Spin RNA II columns (Machery-Nagel Inc., Easton, PA USA). First strand cDNA was synthesized from RNA using random hexamer and oligo dT priming and moloney murine leukemia virus reverse transcriptase (MMLV, Life Technologies) to produce random primed cDNA. Quantitative PCR measurements were performed using the SA Bioscience SYBR Green Supermix (QIAGEN, Valencia, CA USA) reagents on an iCycler Real-Time PCR Detection System (Bio-Rad Inc., Hercules, CA USA). Each reaction was carried out in 15 μl reaction volumes of SA Bioscience SYBR Green Supermix with 5 pM of each primer and diluted first-strand cDNA. All cDNA samples were measured in duplicate. Ribosomal protein large protein 38 (*Rplp38)* was used as the housekeeping gene control for *ΔC_T_* calculation [*ΔC_T_*  =  (*C_T_* of the target gene) – (average *C_T_* of housekeeping genes)]. Primer sequences were listed in Table S2. Fold change expression values were calculated using 2^−ΔΔCT^ methods[57], where ΔΔC_T_  =  (*ΔC_T_* of the treatment sample) – (*ΔC_T_* of control treatment samples) with no added IGF1 as control treatment and normalized to 1.

### Primary Myoblast Isolation and Culture

Primary myoblasts were isolated from hind limb skeletal muscles at 3–5 weeks of age. Muscles were washed with Dulbecco's Phosphate-Buffered Saline (DPBS), minced and digested in type I collagenase and dispase B mixture (Roche Applied Science, Indianapolis, IN USA). The digested muscle pulp was then filtered through a 100 μm filter (CellTrics®, Partec Inc., Swedesboro, NJ USA) to remove large muscle fiber debris and then plated on culture dishes. In 3 days, the cells together with the small debris were collected and digested with 0.025% trypsin for 10 minutes with agitation. Cells were seeded in growth media (F-10 Ham's medium supplemented with 20% fetal bovine serum, 100 units of penicillin, 100 μg of streptomycin,0. 292 mg/ml of L-glutamine, and 4 ng/mL basic fibroblast growth factor) on rat-tail collagen (Roche Applied Science)-coated dishes with preplating on non-coated plates for 45 minutes to deplete fibroblasts as previous described [15], [58]. Myoblasts were differentiated into myotubes after plating cells at approximately 80% confluency on Matrigel (BD Biosciences, San Jose, CA USA) coated plates and the addition of fusion media consisting of DMEM supplemented with 5% horse serum, 100 units of penicillin, 100 μg of streptomycin, and 0.292 mg/ml of L-glutamine. Long®R3 IGF1 (Sigma-Aldrich Co, St Louis, MO USA) was added to the fusion medium for 15 minutes at 3 days after differentiation. Cells were collected for analysis of protein expression.

### Protein Extraction and Western Blots Analysis

*Semimembranosus* and *supraspinatus* muscles were extracted from callipyge and normal lambs at 30–35 days. Samples were frozen immediately after dissection and homogenized in RIPA buffer [50 mM Tris-HCl, 150 mM NaCl, 2 mM EDTA, 10% TritonX100, 0.5% Deoxycholate, 0.1% SDS and 1 × complete protease inhibitor (Roche Applied Science)]. Samples were centrifuged at 10,000 × g for 10 minutes at 4°C. The supernatant was transferred into ultracentrifuge tubes and centrifuged at 50,000 × g for 15 minutes at 4°C.

Proteins were quantified by BCA assay (Thermo Scientific, Rockford, IL USA) per manufacturer's recommendation. A total of 60 μg of protein from each muscle sample was diluted in SDS loading buffer, denatured by heating for 5 minutes in boiling water and loaded in the 6%–18% gradient SDS-PAGE gel. The gel was transferred to PVDF membrane by semi-dry transfer using 20% methanol, 40 mM glycine, 0.15% ethanolamine, 0.2% SDS at 180 mA for 1 h 45 min. PVDF membrane was blocked overnight with agitation in 5% sodium caseinate in PBS with 0.1% Tween 20. Antibodies used for blotting were goat polyclonal anti-Park7 (sc-27006, Santa Cruz Biotechnology, Santa Cruz, CA USA), anti-bovine DLK1 (provided by Dr. Ross L. Tellam, CSIRO, St. Lucia, QLD Australia), mouse monoclonal anti-tubulin (G67, Developmental Studies Hybridoma Bank, Iowa City IA USA) and secondary antibodies were rabbit anti-goat antibody conjugated HRP, goat anti-rabbit conjugated HRP, or goat anti-mouse conjugated HRP respectively (Thermo Scientific Pierce, Rockford, IL USA). The blots were incubated in primary antibody 2 hours at room temperature with a dilution of 1∶1000. After washing 3 times with DPBS, the blots were incubated with secondary antibody with dilution of 1∶20000. Super Signal West Pico Chemiluminescent Substrate (Thermo Scientific Pierce) was used to detect HRP on immunoblots. The blots were visualized using Gel Logic 2200 PRO (Carestream Molecular Imaging System) with exposure times of 2–6 minutes. Carestream Molecular Imaging Software was used to quantify the chemiluminescent signal intensity in arbitrary units. The signal intensities for PARK7 were compared using a t-test without normalization.

### Analysis of Myotube Differentiation and Size

Primary myotubes were fixed in 4% formaldehyde and permeabilized in 0.5% Triton X-100. Cells were blocked in 1% bovine serum albumin (BSA) in PBS for 1 hour at room temperature and stained with 1∶1000 anti-myosin heavy chain antibody (MF20, Developmental Studies Hybridoma Bank, Iowa City, IA USA) for 2 hours. Cells were washed in PBS, and then incubated in 1∶1000 Alexa Fluor 594-conjugated donkey anti-mouse IgG secondary antibodies (Life Technologies) for 1 hour. Finally, cells were mounted with Prolong Gold antifade reagent with DAPI (Life Technologies). The IP Lab software (Scanalytics Inc., Madison, WI USA) and Leica DM6000 microscope were used to acquire pictures.

The fusion index was defined as the ratio of nuclei within the myotubes (>2 nuclei) to the total number of nuclei. The average number of nuclei per myotube was determined by counting over 500 nuclei from randomly chosen MHC-positive cells. Myotube diameters were measured at 5 points along the entire tube. A total of 150 myotubes were examined for each genotype in the experiment.

### *In situ* ELISA Quantification of Myosin Expression

Primary myoblasts were put into Matrigel coated-96-well plate at a high density with four replicate wells for each genotype by IGF1 treatment combination. The myoblasts were fused into myotubes for 3 days in fusion media with different concentration (0, 12.5, 25, 50, 100 and 200 ng/mL) of IGF1. The mature myotubes were fixed in 4% formaldehyde in PBS for 5 minutes at room temperature, washed 3 times with PBS and permeabilized with 0.5% TritonX100 for 5 minutes. After washing 3 times with PBS, the cells were blocked for 1 hour in 1% BSA in PBS. Cells were incubated with anti-myosin heavy chain antibody (MF20, Developmental Studies Hybridoma Bank) diluted in 0.1% BSA overnight at 4°C, and washed 5 times in PBS. Secondary antibody (Donkey anti-mouse IgG, Life Technologies) were diluted in 0.1% BSA and filtered through 0.2 μM filters. Cells were incubated in secondary antibody for 1 hour and washed 5 times with PBS. The fluorescence signals were read on Tecan Genios Pro (Tecan Group Ltd., Morrisville, NC USA) plate reader.

### AKT Phosphorylation in Primary Myotubes

Cultured primary myoblasts or myotubes were used to examine AKT phosphorylation. Different concentrations of IGF1 were added into culture for 15 minutes to induce phosphorylation of AKT. For the PTEN inhibitor experiment, different concentrations (0, 100 nM and 800 nM) of VO-OHpic trihydrate (Sigma-Aldrich) were added into culture and incubated with myotubes for 15 minutes after removal of IGF1. For testing sustained activity of phosphorylation of AKT, IGF1 was removed after 15 minutes incubation with myotubes. The cells were incubated in normal differentiation conditions and cell lysis was collected at the indicated time points (0, 45, 90, 180, 360, 720 minutes). Cells attached to plates were rinsed with ice-cold DPBS, and scraped on ice in cell lysis buffer (50 mM Tris, pH 7.5, 150 mM NaCl, 20 mM sodium fluoride, 1% Triton 100, 1 X phos-stop (Roche Applied Science) and 1 X complete protease inhibitor cocktail. Cells were collected in 1.5 mL microcentrifuge tubes, rotated for 45 minutes, and centrifuged at 12,000 × g for 10 minutes at 4°C to remove insoluble debris.

A total of 45 μg of protein from each cell sample was diluted in SDS loading buffer, and loaded in the 6%–18% gradient SDS-PAGE gel. The gel electrophoresis and transfer to PVDF membranes were the same as described above. The PVDF membranes were blocked in 5% non-fat milk in Tris-Buffered Saline (TBS) with 0.1% Tween 20 for 3 hours. Antibodies used for blotting were rabbit polyclonal anti-AKT (Cat #: 4685, Cell Signaling Technology, Danvers, MA USA), anti-phospho-AKT (Ser473) (Cat #: 4695 Cell Signaling Technology), anti-PTEN (Cat #: 9188 Cell Signaling Technology). The primary antibody was incubated at 4°C overnight at a dilution of 1∶1000. The goat anti-rabbit conjugated HRP was used as secondary antibody and was incubated for 1 hour at 4°C at a dilution of 1∶20000. The visualization of blots was the same as described above.

### Statistical Analysis Methods

All of the statistical analysis presented in this work was conducted using SAS 9.2 software (Statistical Analysis Systems Institute, Cary NC, USA) with assistance from the Statistical Consulting Service at Purdue University. The linear regression models for pairwise comparisons of the three *Park7* genotypes used in the mouse growth trials are provided in Supplementary Table S1. Assays that compared two genotypes, including the western blot data for sheep skeletal muscle (Figure 1), the myotube fusion index (Figure 2B) and myotube diameters (Figure 2C) used a t-test. The in situ ELISA (Figure 4) and qPCR analysis (Figures 5 and 7B) used a two-way analysis of variance with Park7 genotype and IGF1 treatment as main effects along with the genotype by IGF1 interaction. The main effects and interaction statistics are shown with the figures.

## Supporting Information

Figure S1 **Analysis on live body weight by age in** ***Park7*** **(+/+), (+/-) and (-/-) mice.** Twenty females with 4 *Park7* (+/+), 12 (+/-) and 4 (-/-) animals and twenty-five males with 4 *Park7* (+/+), 11 (+/-) and 10 (-/-) were examined to analyze the live animal growth. Animals live body weights were collected from 3 weeks to 6 weeks old. Live body weights were regressed on age. Equations were given for each genotype. There were no genotype effect on the slopes and intercepts of any regression lines in both male and female.
(DOCX)

Table S1 **Regression analysis of growth in Park7 (+/+), (+/-) and (-/-) mice.**
(DOCX)

Table S2 **Quantitative PCR primers.**
(DOCX)

Checklist S1 (DOC)
