# Supplementary material for: Park7 Expression Influences Myotube Size and Myosin Expression in Muscle
Source: PLoS One. 2014 Mar 17;9(3):e92030. doi: 10.1371/journal.pone.0092030 (PMC3956870; doi:10.1371/journal.pone.0092030)
Supplement: Table S1 — Regression analysis of growth in Park7 (+/+), (+/-) and (-/-) mice. (DOCX) [file pone.0092030.s002.docx]

| Table S1. Regression analysis of growth in *Park7* (+/+), (+/-) and (-/-) mice. | | | | |
| --- | --- | --- | --- | --- |
| General Regression Model: | | | | |
| Y = β_0_X+ β_1_*geno1X + β_2_*geno2X + β_3_ + β_4_ *geno1+ β_5_ *geno2 | | | | |
| Coded Variables | Genotypes | Regression Models by Genotype | | |
| geno1 = 1 | +/+ | y = (β_0_ + β_1_)X + (β_3_ + β_4_) | | |
| geno2 = 0 |  |  |  |  |
| geno1 = 0 | +/- | y = (β_0_ + β_2_)X + (β_3_ + β_5_) | | |
| geno2 = 1 |  |  |  |  |
| geno1 = 0 | -/- | y = β_0_X + β_3_ | | |
| geno2 = 0 |  |  |  |  |
|  |  |  | | |
| Males: |  | Regression Equations | | |
| Live weight (g)  by age (weeks) | +/+ | y = 4.4442*age - 3.5735 | | |
|  | +/- | y = 4.0585*age - 2.1310 | | |
|  | -/- | y = 4.1782*age - 2.9579 | | |
| Carcass(g) by  live weight(g) | +/+ | y = 0.6009*lw - 5.3759 | | |
|  | +/- | y = 0.0191*lw + 7.0533 | | |
|  | -/- | y = 0.8254*lw - 9.8533 | | |
| Heart(g) by  live weight(g) | +/+ | y = 0.0042*lw + 0.0195 | | |
|  | +/- | y = 0.0017*lw + 0.0722 | | |
|  | -/- | y = 0.0087*lw - 0.0750 | | |
| Liver(g) by  live weight(g) | +/+ | y = 0.0637*lw - 0.5389 | | |
|  | +/- | y =-0.0094*lw + 1.0736 | | |
|  | -/- | y =-0.0733*lw + 2.4782 | | |
| Kidney(g) by  live weight(g) | +/+ | y = 0.0127*lw - 0.0277 | | |
|  | +/- | y = 0.0176*lw - 0.1177 | | |
|  | -/- | y = 0.0116*lw + 0.0265 | | |
|  |  | live weight (lw) | | |
|  |  |  | | |
|  |  | P-Values | | |
|  | Contrasts | Slope |  | Intercept |
| Live weight | +/+ vs -/- | 0.6719 |  | 0.8858 |
|  | +/- vs -/- | 0.8299 |  | 0.7058 |
|  | +/+ vs +/- | 0.5521 |  | 0.6650 |
| Carcass | +/+ vs -/- | 0.6852 |  | 0.6696 |
|  | +/- vs -/- | 0.1328 |  | 0.1350 |
|  | +/+ vs +/- | 0.0951 |  | 0.0970 |
| Heart | +/+ vs -/- | 0.5111 |  | 0.5135 |
|  | +/- vs -/- | 0.2997 |  | 0.3015 |
|  | +/+ vs +/- | 0.5645 |  | 0.5520 |
| Liver | +/+ vs -/- | 0.2065 |  | 0.2268 |
|  | +/- vs -/- | 0.5347 |  | 0.5559 |
|  | +/+ vs +/- | 0.2841 |  | 0.3015 |
| Kidney | +/+ vs -/- | 0.8721 |  | 0.9447 |
|  | +/- vs -/- | 0.6610 |  | 0.7022 |
|  | +/+ vs +/- | 0.6757 |  | 0.6280 |

| Table S1 Continued | | | | |
| --- | --- | --- | --- | --- |
|  |  |  | | |
| Females: | Genotypes | Regression Equations | | |
| Live weight (g)  by age (weeks) | +/+ | y = 2.9658* age + 0.1472 | | |
|  | +/- | y = 2.8799* age + 0.8168 | | |
|  | -/- | y = 3.4308* age - 3.1265 | | |
| Carcass(g) by  live weight(g) | +/+ | y = 0.6396* lw - 4.2095 | | |
|  | +/- | y = 0.6389* lw - 4.7451 | | |
|  | -/- | y = 0.3022* lw + 0.3916 | | |
| Heart (g) by  live weight(g) | +/+ | y = 0.0025* lw + 0.0504 | | |
|  | +/- | y = 0.0050* lw + 0.0055 | | |
|  | -/- | y = 0.0025* lw + 0.0465 | | |
| Liver (g) by  live weight(g) | +/+ | y = 0.0737* lw - 0.4577 | | |
|  | +/- | y = 0.0744* lw - 0.5963 | | |
|  | -/- | y =-0.0260* lw + 0.2008 | | |
| Kidney(g) by  live weight(g) | +/+ | y = 0.0144* lw - 0.0370 | | |
|  | +/- | y = 0.0159* lw - 0.0749 | | |
|  | -/- | y = 0.0137* lw - 0.0373 | | |
|  |  |  | | |
|  |  | P-Values | | |
|  | Contrasts | Slope |  | Intercept |
| Live weight | +/+ vs -/- | 0.5108 |  | 0.8757 |
|  | +/- vs -/- | 0.5597 |  | 0.1799 |
|  | +/+ vs +/- | 0.1778 |  | 0.1520 |
| Carcass | +/+ vs -/- | 0.4477 |  | 0.3620 |
|  | +/- vs -/- | 0.4126 |  | 0.3721 |
|  | +/+ vs +/- | 0.8991 |  | 0.9977 |
| Heart | +/+ vs -/- | 0.9734 |  | 0.9962 |
|  | +/- vs -/- | 0.7314 |  | 0.7215 |
|  | +/+ vs +/- | 0.5855 |  | 0.5869 |
| Liver | +/+ vs -/- | 0.4620 |  | 0.3820 |
|  | +/- vs -/- | 0.3907 |  | 0.3848 |
|  | +/+ vs +/- | 0.8245 |  | 0.9855 |
| Kidney | +/+ vs -/- | 0.9985 |  | 0.9353 |
|  | +/- vs -/- | 0.7949 |  | 0.8044 |
|  | +/+ vs +/- | 0.7031 |  | 0.7994 |
