# Supplementary material for: Park7 Expression Influences Myotube Size and Myosin Expression in Muscle
Source: PLoS One. 2014 Mar 17;9(3):e92030. doi: 10.1371/journal.pone.0092030 (PMC3956870; doi:10.1371/journal.pone.0092030)
Supplement: Table S2 — Quantitative PCR primers. (DOCX) [file pone.0092030.s003.docx]

| Gene name | Forward primer (5’-3’) | Reverse primer (5’-3’) |
| --- | --- | --- |
| *Myh7* | AGTCCCAGGTCAACAAGCTG | TTCCACCTAAAGGGCTGTTG |
| *Myh3* | CGCAGAATCGCAAGTCAATA | ATATCTTCTGCCCTGCACCA |
| *Myh8* | AGTCCCAGGTCAACAAGCTG | CCTCCTGTGCTTTCCTTCAG |
| *Myh4* | AGTCCCAGGTCAACAAGCTG | TTTCTCCTGTCACCTCTCAACA |
| *Pten* | CCTTTTGAAGACCATAACCCACC | GAATTGCTGCAACATGATTGTCA |
| *Rplp38* (control) | GAAGGATGCCAAGTCTGTCAA | GAGGGCTGGTTCATTTCAGA |
