# Supplementary material for: Park7 Expression Influences Myotube Size and Myosin Expression in Muscle
Source: PLoS One. 2014 Mar 17;9(3):e92030. doi: 10.1371/journal.pone.0092030 (PMC3956870; doi:10.1371/journal.pone.0092030)
Supplement: Supplementary file 4 [file pone.0092030.s001.docx]

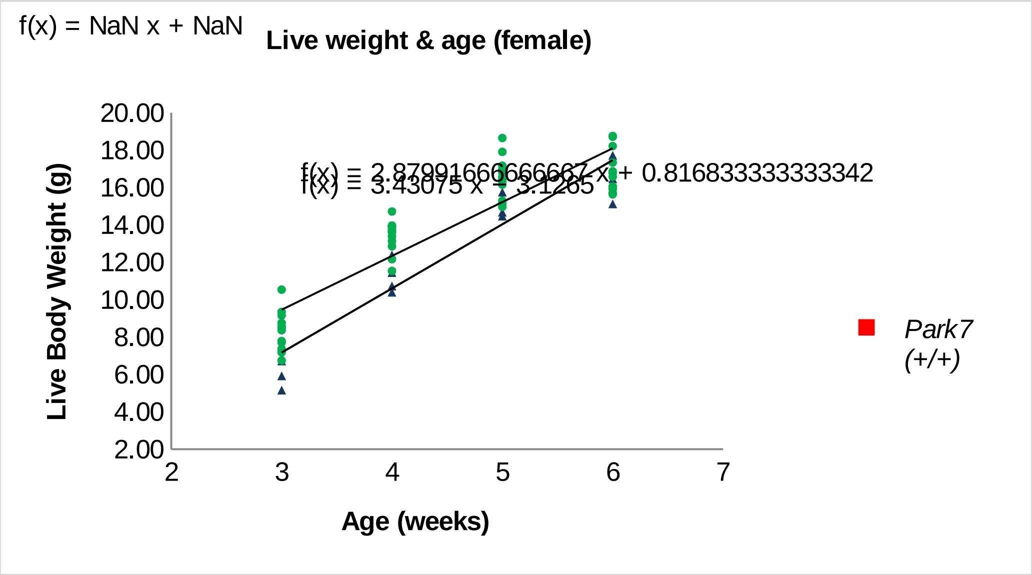

f(x) = NaN x + NaN
Live weight & age (female)
f(x) = 2.87991666666667 x + 0.816833333333342
f(x) = 3.43075 x − 3.1265
Live Body Weight (g)
20.00
18.00
16.00
14.00
12.00
10.00
8.00
6.00
4.00
2.00
2
3
4
5
6
7
Age (weeks)
Park7 (+/+)

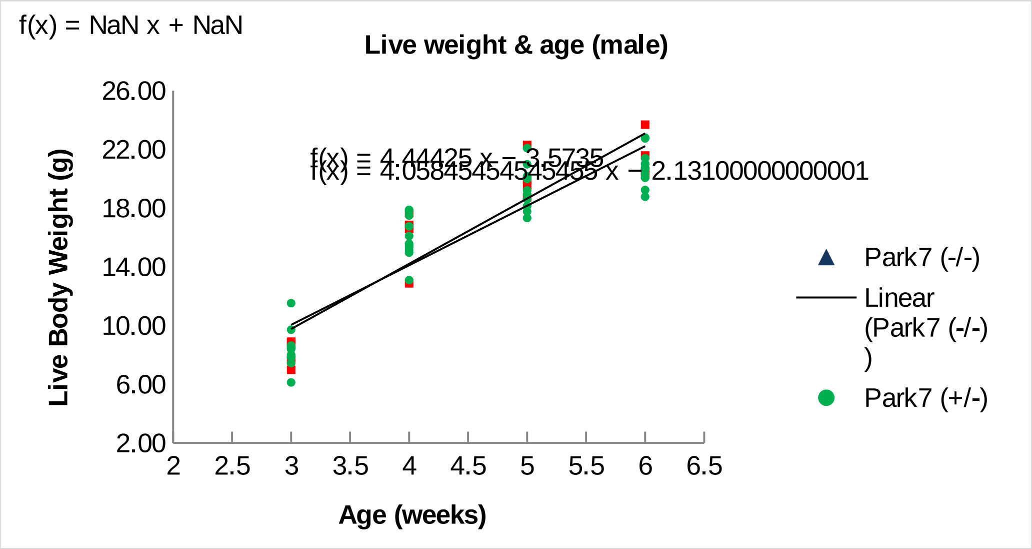

f(x) = NaN x + NaN
Live weight & age (male)
f(x) = 4.44425 x − 3.5735
f(x) = 4.05845454545455 x − 2.13100000000001
Live Body Weight (g)
26.00
22.00
18.00
14.00
10.00
6.00
2.00
2
2.5
3
3.5
4
4.5
5
5.5
6
6.5
Age (weeks)
Park7 (-/-)
Linear (Park7 (-/-))
Park7 (+/-)
